# Supplementary material for: TB Antigen-Stimulated CXCR3 Ligand Assay for Diagnosis of Tuberculous Lymphadenitis
Source: Int J Environ Res Public Health. 2021 Jul 29;18(15):8020. doi: 10.3390/ijerph18158020 (PMC8345678; doi:10.3390/ijerph18158020)
Supplement: Supplementary file 1 [file ijerph-18-08020-s001.zip › ijerph-1267217-supplementary.pdf]

As advised, in the KU analysis, p values of both significant and insignificant results are informative in that they comparatively represent the degree of significances, and can also show a trend toward significance. We have presented all the exact p values of Dunn's comparison test as below. We suppose that it would be more appropriate to present the p value data as supplements because we are afraid that the figures might be too complicated if all the p values would be indicated directly in the figures (i.e.; for the sake of clarity and visibility).

From the table of precise p values, it is clearly shown that I-TAC has the most significant differences compared to all other groups. With regard to differentiating TB-LAP patients from LTBI, I-TAC showed significant results in both all and definite patient groups, while IFN-γ showed only a trend toward significant difference in all TB-LAP patients (p = 0.099), and MIG showed only a trend toward significant difference (p = 0.010) in definite TB-LAP patients.

Table S1. The significance levels of pairwise comparisons among all TB-LAP patients, Non-TB-LAP patients, IGRA$^+$ controls, and IGRA$^-$ controls.

P values	TB-LAP	Non-TB-LAP	IGRA$^+$ controls	IGRA$^-$ controls
IFN-γ				
TB-LAP		0.000385	0.099222	<0.000001
Non-TB-LAP			0.224845	0.215273
IGRA$^+$ controls				<0.000001
IGRA$^-$ controls				
I-TAC				
TB-LAP		0.000017	0.000123	<0.000001
Non-TB-LAP			0.872838	1
IGRA$^+$ controls				0.001672
IGRA$^-$ controls				
MIG				
TB-LAP		0.004633	0.007725	<0.000001
Non-TB-LAP			1	0.035846
IGRA$^+$ controls				<0.000001
IGRA$^-$ controls				

Numbers are p values for each pairwise comparison (Kruskal Wallis analysis followed by Dunn's comparison test).
TB-LAP = tuberculous lymphadenitis; Non-TB-LAP = non-tuberculous lymphadenitis; IGRA = interferon-γ release assay; IGRA$^+$ controls = IGRA positive controls; IGRA$^-$ controls = IGRA

negative controls; IFN-γ = interferon-γ; I-TAC = interferon-inducible T cell chemoattractant; MIG = monokine induced by interferon-γ

Table S2. The significance levels of pairwise comparisons among definite TB-LAP patients, Non-TB-LAP patients, IGRA^{+} controls, and IGRA^{-} controls.

P values	TB-LAP	Non-TB-LAP	IGRA^{+} controls	IGRA^{-} controls
IFN-γ				
TB-LAP		0.001074	0.22795	<0.000001
Non-TB-LAP			0.189065	0.214593
IGRA^{+} controls				<0.000001
IGRA^{-} controls				
I-TAC				
TB-LAP		0.000011	0.000125	<0.000001
Non-TB-LAP			0.63741	1
IGRA^{+} controls				0.001212
IGRA^{-} controls				
MIG				
TB-LAP		0.008033	0.010391	<0.000001
Non-TB-LAP			1	0.035868
IGRA^{+} controls				<0.000001
IGRA^{-} controls				

Numbers are *p* values for each pairwise comparison (Kruskal Wallis analysis followed by Dunn's comparison test).

TB-LAP = tuberculous lymphadenitis; Non-TB-LAP = non-tuberculous lymphadenitis; IGRA = interferon-γ release assay; IGRA^{+} controls = IGRA positive controls; IGRA^{-} controls = IGRA negative controls; IFN-γ = interferon-γ; I-TAC = interferon-inducible T cell α chemoattractant; MIG = monokine induced by interferon-γ
